# Multimorbidity is significantly associated with higher prevalence of depressive symptoms in middle-aged and older Chinese adults

**DOI:** 10.1016/j.pmedr.2025.103289

**Published:** 2025-10-30

**Authors:** Yiyang Zheng, Pengyao Lin, Liming Zhao, Tianchen Qian, Jiarong Xie, Lei Xu

**Affiliations:** a Gastroenterology Department, The First Affiliated Hospital of Ningbo University, Ningbo 315010, China; b Department of Gastroenterology, The First Affiliated Hospital, College of Medicine, Zhejiang University, Hangzhou 310003, China

**Keywords:** Depressive symptoms, Multimorbidity, Chronic diseases, CESD-10, China

## Abstract

**Objective:**

Chronic diseases and depressive symptoms are prevalent global health concerns. This study examined the association between multimorbidity and depressive symptoms in the middle-aged and older adults.

**Methods:**

This cross-sectional study used data from the China Health and Retirement Longitudinal Study (CHARLS, China, 2011–2020), including 18,551 adults aged ≥45 years. Depressive symptoms were assessed with the 10-item Center for Epidemiologic Studies Depression Scale (score ≥10). We analyzed associations for each chronic disease and for their coexistence.

**Results:**

After adjusting for sociodemographic and lifestyle factors, chronic liver disease, cardiovascular disease, chronic kidney disease, and diabetes were significantly associated with depressive symptoms. Participants with ≥2 of these conditions had higher odds of depressive symptoms (OR = 2.34, 95 % CI: 1.89, 2.89). Subgroup analyses showed consistent associations within strata. In age-stratified analyses, adjusted ORs were 2.96 for participants <60 years and 2.26 for those ≥60 years. In sex-stratified analyses, adjusted ORs were 2.26 for females and 2.70 for males. In body mass index analyses (BMI), adjusted ORs were 3.60 for <24 kg/m^2^ and 1.88 for ≥24 kg/m^2^.

**Conclusions:**

Multimorbidity was significantly associated with depressive symptoms among Chinese adults aged ≥45 years. These associations were consistent in sex, age, and BMI.

## Highlights

• Depressive symptoms linked to chronic diseases and rose with multimorbidity.
• Multimorbidity related to depressive symptoms in middle-aged and older adults.
• Multimorbidity was associated with depressive symptoms in both males and females.

## 1 Introduction

As populations age, chronic diseases have become a major challenge for global public health, accounting for approximately 41 million deaths annually and representing 74 % of all deaths worldwide (Organization, 2023). Cardiovascular disease (CVD) is highly prevalent and commonly co-occurs with chronic liver disease, chronic kidney disease, and diabetes in middle-aged and older adults (Li et al., 2021). More than 60 % of patients present with multiple chronic conditions simultaneously (Spiers et al., 2019). The cardiovascular–kidney–metabolic (CKM) syndrome was introduced to describe this frequent coexistence of cardiovascular, kidney, and metabolic disorders. It summarizes clinical observations that these conditions often occur together, share overlapping risk factors, and have been associated with higher health burden in middle-aged and older adults (Ndumele et al., 2023). Such clustering of diseases has implications beyond physical health, as multimorbidity may amplify both physical and psychological burdens. When these conditions coexist, they are not only important clinical outcomes but also essential contextual factors for understanding mental health (Tazzeo et al., 2023; Xie et al., 2025).

Depressive symptoms, a common mental disorder, are widespread among middle-aged and older adults. The prevalence rate is 35.1 % (World Health Organization. Regional Office for the Eastern, 2019). They may be associated with chronic diseases, prevalent in middle-aged and older adults, hich could further contribute to depressive symptoms (Ai et al., 2023; Zheng et al., 2024).

Previous studies have provided initial evidence for associations between depressive symptoms and chronic diseases, including diabetes and cardiovascular disease. Several of these studies have focused on Western populations (Khawagi et al., 2024; Meng et al., 2020). Using data from the China Health and Retirement Longitudinal Study (CHARLS) (Zhao et al., 2014), we extended prior work by examining associations between depressive symptoms and four chronic diseases in Chinese adults aged ≥45 years, namely chronic liver disease, cardiovascular disease, chronic kidney disease, and diabetes. Analyses were conducted separately for each disease and for the co-occurrence of two or more conditions, thereby extending previous research by distinguishing disease-specific associations from those reflecting multimorbidity.

Sociodemographic and lifestyle characteristics represent potential sources of confounding, as they are linked to both chronic disease occurrence and depressive symptoms (Bobo et al., 2022; Sigvardsen et al., 2025). Adjusting for these variables is therefore important to ensure valid estimation of the associations of interest.

Analyses were stratified by age, sex, and BMI to examine whether the associations between chronic diseases and depressive symptoms varied across demographic and metabolic subgroups. Age stratification was applied given potential variation in the prevalence and impact of both chronic diseases and depressive symptoms across midlife and older adulthood (Triolo et al., 2023). Regarding sex, depressive symptoms are more frequently observed in females (Li et al., 2023). However, whether the relationship between chronic diseases and depressive symptoms differs between males and females remains unclear, prompting sex-stratified analyses. BMI stratification enabled comparisons across weight categories, as adiposity and metabolic status have been implicated in modifying the relationship between chronic diseases and depressive symptoms (Jokela and Laakasuo, 2023). These stratified analyses aimed to explore potential subgroup variations that could inform more tailored prevention and management strategies for depression among individuals with chronic diseases.(Richmond-Rakerd et al., 2021).

## 2 Methods

### 2.1 Research design and participants

**Fig. 1:**
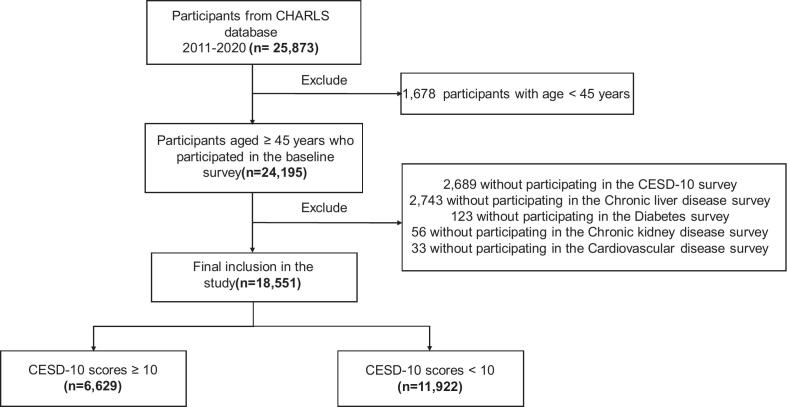
Flow diagram of participant selection in the China Health and Retirement Longitudinal Study, 2011–2020. CHARLS, China Health and Retirement Longitudinal Study; CESD-10, Center for Epidemiologic Studies Depression Scale-10.

Data for this study were obtained from the CHARLS (2011–2020), a nationally representative survey of Chinese adults aged ≥45 years (Zhao et al., 2014). A total of 5644 individuals were excluded due to the following reasons: (1) 2689 participants did not complete the 10-item Center for Epidemiological Studies Depression Scale (CESD-10) survey; (2) chronic liver disease survey data were not available for 2743 individuals; (3) diabetes survey was not completed for 123 individuals; (4) chronic kidney disease survey was missing for 56 individuals; (5) cardiovascular disease survey was missing for 33 individuals. Accordingly, 18,551 individuals were finally included (Fig. 1). The CHARLS survey employed structured face-to-face interviews conducted by trained staff, using standardized questionnaires to collect information on health status, lifestyle, socioeconomic conditions, and physician-diagnosed chronic diseases. The CHARLS study received approval from the Biomedical Ethics Committee at Peking University (Approval Number: IRB00001052-11015), and written informed consent was obtained from all participants.

### 2.2 Chronic diseases

Diseases were diagnosed by asking participants if they had ever been informed by a healthcare professional that they had a chronic disease. The diseases mainly included in this list were diabetes (both type 1 and type 2 diabetes) (Yang et al., 2024), chronic liver disease (viral hepatitis, alcoholic liver disease, autoimmune liver disorders, genetic liver conditions, drug or toxin-induced liver damage, and cirrhosis) (Balakrishnan and Rehm, 2024), cardiovascular disease (heart attack, coronary heart disease, angina pectoris, congestive heart failure or other cardiac problems) (Martin et al., 2024), chronic kidney disease (glomerular diseases, tubular diseases, interstitial diseases, and vascular diseases) (Liang and Liu, 2023). Additionally, multimorbidity refers to when two or more of these diseases coexist.

### 2.3 Depressive symptoms

In this study, the CESD-10 scale was used to assess depressive symptoms. This scale has been widely validated in Chinese populations (Cosco et al., 2020; Ren et al., 2023). An adapted version of the CESD also measures depressive symptoms and has demonstrated sound psychometric properties (Zebrowska et al., 2024). Items were scored from 0 to 3, with the fifth and eighth items reverse coded. Depressive symptoms can be determined when the total score of the scale reaches or exceeds 10 points. The CESD-10 scale is reliable and widely used to screen for depressive symptoms (Cosco et al., 2020).

### 2.4 Statistical analyses

Categorical variables are presented as percentages, and group differences were assessed using the χ^2^ test. Continuous variables with a normal distribution are reported as the mean ± standard deviation (SD), while those with a non-normal distribution are expressed as the median and interquartile range (IQR).

The study encompassed covariates such as age, sex (male, female), smoking history, alcohol consumption history, body mass index (BMI), marital status, educational levels (less than primary, primary, secondary, high school and above), total household income, chronic liver disease, cardiovascular disease, chronic kidney disease, and diabetes.

Logistic regression analysis was employed to explore the association between chronic diseases and the presence of depressive symptoms. Several covariates were incorporated into two models as follows: (1) Unadjusted model; (2) Fully adjusted model, which adjusted for demographic factors (age and sex) and sociobehavioral and anthropometric variables, including smoking status, alcohol consumption, marital status, education level, household income, and BMI. These variables were collectively included in the fully adjusted model because socioeconomic position, lifestyle behaviors, and body weight are interrelated determinants influencing both chronic diseases and depressive symptoms. The odds ratio (OR) and its corresponding 95 % confidence intervals (CIs) were derived from the logistic regression analyses.

Additionally, to assess the robustness of the findings, subgroup analyses were performed to examine the relationship between chronic diseases, depressive symptoms, and study outcomes across different categories, including sex (male, female), BMI (<24 kg/m^2^ and ≥24 kg/m^2^, defined according to population-specific criteria that classify individuals as having normal weight or overweight in Chinese adults) (Wang and Zhai, 2013), and age (< 60 years and ≥60 years) (Lu et al., 2025). The overall cohort was defined as adults aged ≥45 years, and the <60 versus ≥60 years stratification was applied to differentiate middle-aged from older participants. Statistical analyses were carried out using Stata software (version 18.0).

## 3 Results

### 3.1 Demographic characteristics of depressive symptoms

Table 1 presents the baseline characteristics of the study population. The mean age of the 18,551 participants was 58.07 years (SD: 9.65 years), and 53.24 % were male. Participants were classified into two groups based on their CESD-10 scores: those in the non-depressed category (score <10, *n* = 11,922) and those in the depressed category (score ≥10, *n* = 6629). Compared with the non-depressed group, the depressed group contained a larger proportion of females, unmarried individuals, participants with lower educational attainment and lower household income, and those reporting smoking or alcohol consumption (all *P* < 0.05).

**Table 1:** Baseline demographic, socioeconomic, and lifestyle characteristics of middle-aged and older adults in China, 2011–2020.

	CESD-10 score < 10, n(%)	CESD-10 score ≥ 10, n(%)	*P* value
Sex			< 0.01
Male	6346 (53.2)	2577 (38.9)	
Female	5574 (46.8)	4052 (61.1)	
Ever drank alcohol	5098 (43.3)	2385 (36.3)	< 0.01
Ever smoked	4984 (41.8)	2335 (35.2)	< 0.01
Married	10,746 (90.2)	5481 (82.7)	< 0.01
Education level			< 0.01
Less than primary	4444 (37.3)	3670 (55.4)	
Primary	2639 (22.2)	1440 (21.7)	
Secondary	2786 (23.4)	1043 (15.7)	
High school and above	2038 (17.1)	474 (7.2)	
Total household income, yuan	18,480 (3560–43,800)	7700 (1720–25,996)	< 0.01
Age, mean (SD)	58.07 (9.7)	59.90 (9.9)	< 0.01
BMI, kg/m^2^	23.46 (21.2–26.1)	22.76 (20.4–25.5)	0.13
Chronic liver disease	337 (2.8)	324 (4.9)	< 0.01
Cardiovascular disease	1185 (9.9)	1081 (16.3)	< 0.01
Chronic kidney disease	496 (4.2)	542 (8.2)	< 0.01
Diabetes	712 (6.0)	487 (7.4)	< 0.01

CESD-10, 10–item center for epidemiological studies depression screening index; BMI, body mass index; SD, standard deviation.

Continuous variables are expressed as mean ± standard deviation, or as median (interquartile range). Categorical variables are expressed as frequency (percent).

*P* values were obtained from the χ^2^ test for categorical variables and from appropriate parametric or non-parametric tests for continuous variables.

Values for income are presented in Chinese yuan (CNY; 1 yuan ≈ 0.14 US dollars).

CESD-10 scores <10 indicate non-depressive symptoms, and scores ≥10 indicate presence of depressive symptoms.

### 3.2 Association between chronic diseases and depressive symptoms

Table 2 presents the association between chronic diseases and depressive symptoms. In the unadjusted model, patients with chronic liver disease exhibited greater odds of depressive symptoms (OR = 1.77, 95 % CI: 1.51, 2.06), as did those with cardiovascular disease (OR = 1.77, 95 % CI: 1.62, 1.93), chronic kidney disease (OR = 2.05, 95 % CI: 1.81, 2.33), and diabetes (OR = 1.25, 95 % CI: 1.11, 1.41). Moreover, multimorbidity was associated with increased odds of depressive symptoms (OR = 2.16, 95 % CI: 1.87, 2.48). Similar results were observed in the fully adjusted model, indicating that these associations remained statistically significant. ORs and 95 % CIs for chronic liver disease, cardiovascular disease, chronic kidney disease, and diabetes were as follows: 2.04 (95 % CI: 1.62, 2.58), 1.73 (95 % CI: 1.52, 1.96), 2.06 (95 % CI: 1.72, 2.48), and 1.36 (95 % CI: 1.14, 1.62), respectively, indicating that these estimates remained statistically significant.

**Table 2:** Logistic regression estimates for associations between chronic diseases and depressive symptoms among middle-aged and older adults in China, 2011–2020.

	Unadjusted model OR (95 % CI)	Fully adjusted model OR (95 % CI)
Chronic liver disease	1.77 (1.51, 2.06)	2.04 (1.61, 2.58)
Cardiovascular disease	1.77 (1.62, 1.93)	1.73(1.52, 1.96)
Chronic kidney disease	2.05 (1.81, 2.33)	2.06 (1.72, 2.48)
Diabetes	1.25 (1.11, 1.41)	1.36 (1.14, 1.62)
Multimorbidity	2.16 (1.87, 2.48)	2.34 (1.89, 2.89)

The fully adjusted model represents the primary analysis based on the DAG-informed covariate set; Unadjusted results are shown for transparency.

OR, odds ratio; 95 % CI, 95 % confidence interval; Multimorbidity, having two or more diseases.

Fully adjusted model, the model was adjusted for age, sex, ever drank alcohol, ever smoked, marital status, education level, total household income.

Furthermore, the OR for multimorbidity remained elevated after adjustment (OR = 2.34, 95 % CI: 1.89, 2.89).

### 3.3 Subgroup analyses

**Fig. 2:**
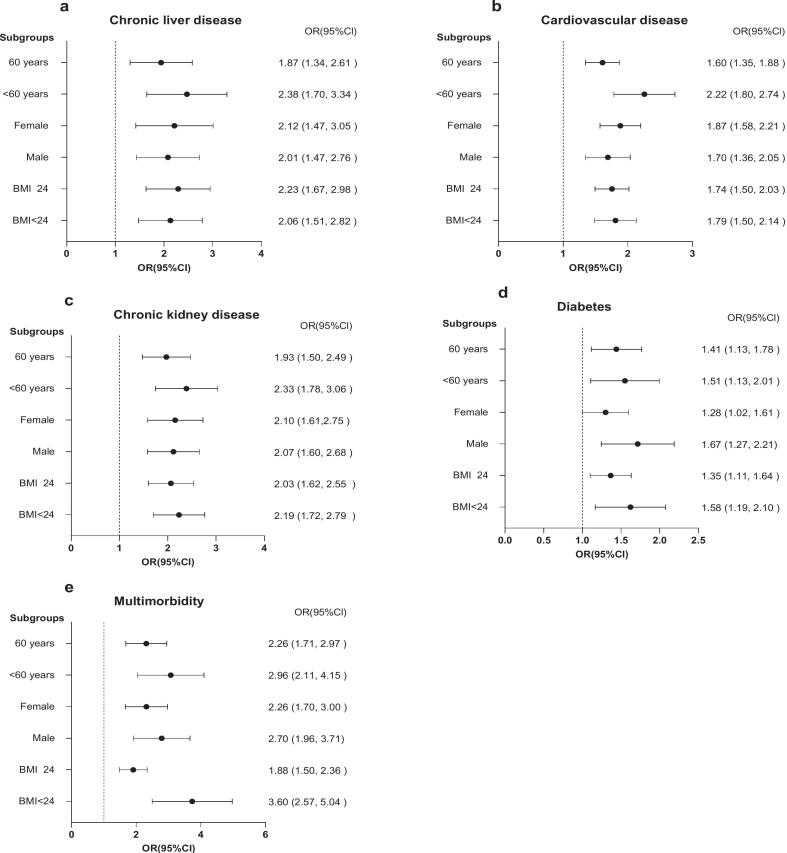
Subgroup-specific associations between chronic diseases and depressive symptoms among middle-aged and older adults in China, 2011–2020. OR, odds ratio; CI, confidence interval; BMI, body mass index.

Subgroup analyses evaluated the association within categories of age (<60 and ≥60 years), sex, and BMI (<24 and ≥ 24 kg/m^2^) (Fig. 2). A significant correlation between chronic disease and depressive symptoms was observed in patients with chronic liver disease aged 60 years or older, with an OR of 1.87 (95 % CI: 1.34, 2.61). The OR increased to 2.38 (95 % CI: 1.70, 3.34) in individuals under 60. The OR for individuals with a BMI of 24 kg/m^2^ or higher was 2.23 (95 % CI: 1.67, 2.98), and for those with a BMI below 24 kg/m^2^ it was 2.06 (95 % CI: 1.51, 2.82). OR was 2.12 (95 % CI: 1.47, 3.05) for female patients, which was slightly higher than that for male patients (OR = 2.01, 95 % CI: 1.47, 2.76).

Among those with cardiovascular disease, patients aged 60 years or older had an OR of 1.60 (95 % CI: 1.35, 1.88), and it was 2.22 (95 % CI: 1.80, 2.74) for patients younger than 60. For female patients, the OR was 1.87 (95 % CI: 1.58, 2.21), whereas for male patients, it was 1.70 (95 % CI: 1.36, 2.05). The OR was 1.74 (95 % CI: 1.50, 2.03) for patients with a BMI of 24 kg/m^2^ or higher, whereas for those with a BMI ≤24 kg/m^2^, it was 1.79 (95 % CI: 1.50, 2.14). Patients with chronic kidney disease and diabetes exhibited similar results, and all findings were significant.

In patients with multimorbidity, the OR for the occurrence of depressive symptoms was observed to be significantly elevated compared to those with a single disease. The OR was 2.26 (95 % CI: 1.71, 2.97) for patients aged 60 years or older, and 2.96 (95 % CI: 2.11, 4.15) for those under 60 years. In terms of sex, the OR was 2.26 (95 % CI: 1.70, 3.00) for female patients and 2.70 (95 % CI: 1.96, 3.71) for male patients. Regarding body mass index (BMI), patients with a BMI of 24 kg/m^2^ or greater had an OR of 1.88 (95 % CI: 1.50, 2.36), while those with a BMI below 24 kg/m^2^ showed an OR of 3.60 (95 % CI: 2.57, 5.04). Statistical significance in age, sex, and BMI strata confirmed that the association between multimorbidity and depressive symptoms was robust in different subgroups.

## 4 Discussion

This study demonstrated that depressive symptoms were significantly associated with major chronic conditions, including chronic liver disease, cardiovascular disease, chronic kidney disease, and diabetes, among middle-aged and older Chinese adults. Moreover, the coexistence of two or more of these conditions, reflecting multimorbidity, further amplified the likelihood of depressive symptoms, suggesting a cumulative burden effect. These disorders are interrelated, and in the literature the term cardiovascular–kidney–metabolic (CKM) syndrome has been proposed to reflect their co-occurrence and clinical interconnections (Ndumele et al., 2023). As simultaneous manifestation of all four conditions is infrequent among middle-aged and older adults, we evaluated those with ≥2 conditions to better capture comorbidity patterns relevant at the population level. The associations were observed consistently in middle-aged and older adults, in females and males, and in both lower and higher BMI groups.

The present analysis extended prior work by using the CHARLS database to investigate associations between depressive symptoms and chronic liver disease, cardiovascular disease, chronic kidney disease, and diabetes, considering both each condition separately and the coexistence of these conditions as multimorbidity (Zuidersma et al., 2011). Multivariable models adjusting for demographic, lifestyle, and socioeconomic factors showed associations between depressive symptoms and each chronic condition examined (chronic liver disease, cardiovascular disease, chronic kidney disease, and diabetes). We further analyzed participants with ≥2 of these conditions concurrently, and the adjusted odds ratio for depressive symptoms exceeded that observed in single-condition analyses (Read et al., 2017). In stratified analyses, the association between multimorbidity and depressive symptoms was observed in both middle-aged (<60 years) and older (≥60 years) adults. Similar associations were also found in females and males and in participants with BMI <24 kg/m^2^ and ≥24 kg/m^2^. These findings demonstrate that the association between multimorbidity and depressive symptoms was consistent in demographic and clinical subgroups, indicating that depressive symptoms should be addressed not only in older adults but also in middle-aged individuals, in both sexes and BMI categories.

Previous studies suggest several potential mechanisms that may be related to these associations, including hepatic inflammation, vascular dysfunction, and insulin resistance with hyperglycemia, which can influence neurovascular and immune regulation (Aburto and Cryan, 2024; Khawagi et al., 2024; Sara et al., 2024). In addition, chronic activation of biological stress systems (Li et al., 2019) may also contribute.

This study had several strengths. First, we examined each chronic disease individually to clarify its specific association with depressive symptoms. We then evaluated their coexistence as multimorbidity, which was associated with a higher prevalence of depressive symptoms. Second, stratified analyses by age, sex, and BMI demonstrated consistent associations, supporting the robustness and generalizability of the findings. Finally, the chronic diseases investigated are interrelated, consistent with the cardiovascular–kidney–metabolic concept that provides a clinical context for interpreting these associations.

There are also some limitations in this study. First, its cross-sectional design limits causal inference and reverse associations cannot be excluded. Second, self-reported data may introduce recall or reporting bias, and residual or unmeasured confounding remains possible. Finally, as the analysis was restricted to a Chinese population, further research is needed to assess generalizability to other groups.

## 5 Conclusions

This study found a significant association between multimorbidity and depressive symptoms in middle-aged and older adults in China. The relationship remained consistent in various sex, age, and BMI categories. These findings suggest that healthcare providers should consider physical and mental health in patients with multimorbidity (Birk et al., 2019; VanderWeele et al., 2020). Future research could use cohort designs to investigate the longitudinal relationship between multimorbidity and depressive symptoms.

## CRediT authorship contribution statement

**Yiyang Zheng:** Writing – review & editing, Writing – original draft. **Pengyao Lin:** Data curation. **Liming Zhao:** Investigation. **Tianchen Qian:** Formal analysis. **Jiarong Xie:** Validation, Conceptualization. **Lei Xu:** Conceptualization.

## Ethics approval

This study was conducted with human participants and received ethical approval from the Biomedical Ethics Committee of Peking University (Approval Number: IRB00001052-11015). Prior to participation, all individuals provided informed consent, ensuring their voluntary involvement in the CHARLS survey project.

## Funding

This work was supported by 10.13039/100005622Ningbo Top Medical and Health Research Program (No. 2023020612) and the Project of Ningbo leading Medical & Healthy Discipline (2022-S04).

## Declaration of competing interest

The authors declare the following financial interests/personal relationships which may be considered as potential competing interests: Lei Xu reports financial support was provided by Ningbo Top Medical and Health Research Program. Lei Xu reports financial support was provided by the project of Ningbo leading Medical & Healthy Discipline. If there are other authors, they declare that they have no known competing financial interests or personal relationships that could have appeared to influence the work reported in this paper.

## Data Availability

The data used in this study are publicly available through an open-access repository. Specifically, the CHARLS datasets analyzed and generated during the current research can be accessed via the official CHARLS website at http://charls.pku.edu.cn/en. All data are de-identified, with respondents distinguished only by unique identification numbers to ensure confidentiality.

## Appendix A Supplementary data

Supplementary material. Directed acyclic graph showing the conceptual framework linking chronic diseases and depressive symptoms among Chinese adults age ≥45 years in the China Health and Retirement Longitudinal Study (2011–2020). Image 1
