# Supplementary figures and images for: Multimorbidity is significantly associated with higher prevalence of depressive symptoms in middle-aged and older Chinese adults
Source: Prev Med Rep. 2025 Oct 30;60:103289. doi: 10.1016/j.pmedr.2025.103289 (PMC12634280; doi:10.1016/j.pmedr.2025.103289)

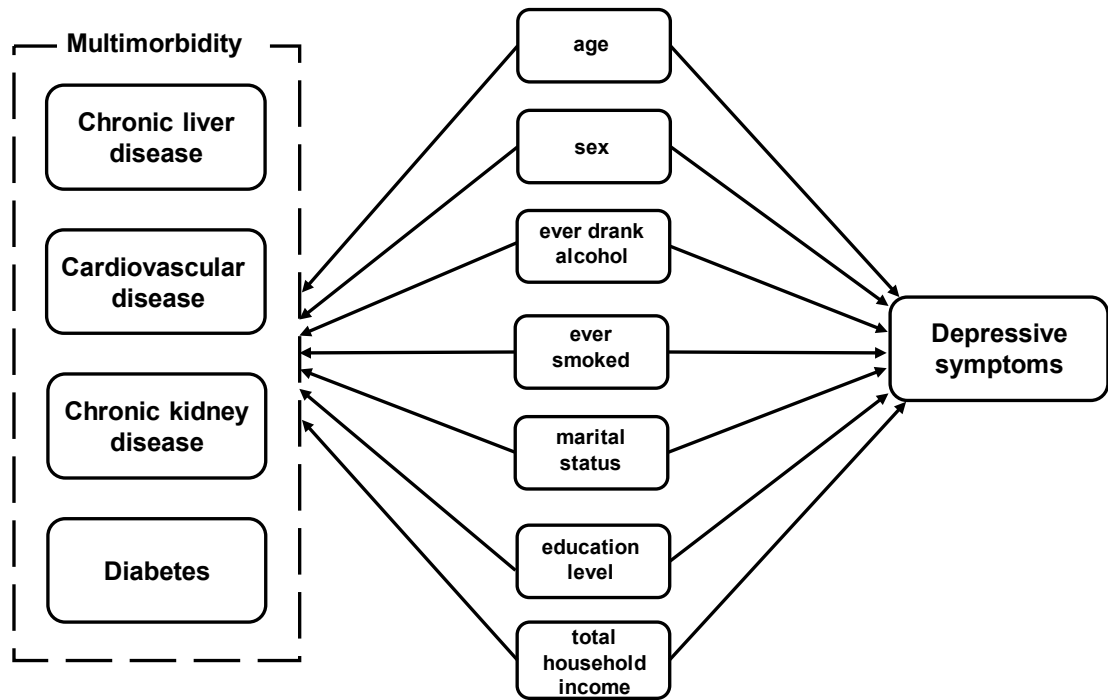

Supplement: Supplementary file 1 — Supplementary material. Directed acyclic graph showing the conceptual framework linking chronic diseases and depressive symptoms among Chinese adults age ≥45 years in the China Health and Retirement Longitudinal Study (2011–2020). [file mmc1.pdf]
